# Synergistic effects of metformin and curcumin on cytotoxicity of chemotherapy drugs using a gastric cancer cell line model

**DOI:** 10.17179/excli2021-4091

**Published:** 2021-10-11

**Authors:** Ehsan Zarei, Youssof Sefidi-Heris, Iraj Saadat

**Affiliations:** 1 Department of Biology, College of Sciences, Shiraz University, Shiraz, Iran

**Keywords:** gastric cancer, metformin, curcumin, chemotherapy, synergistic effect

## Abstract

Gastric cancer has a weak prognosis and its usual treatments depend on surgery and chemotherapy. These treatments suffer from some drawbacks such as high rates of local recurrence and metastasis, low survival rates, and significantly decreased life quality. Therefore, new therapeutic methods for improved gastric cancer care with minimal side effects seem necessary. Currently, combinatorial treatments for cancer are preferred and recently, metformin (Met) and curcumin (Cur) have been interesting options for this type of therapy. The aim of the present study was to investigate anticancer effects of metformin and curcumin in both single and combinatorial treatment forms on AGS gastric cancer cell line. In comparison to single treatments with each substance, the results of co-treatments with metformin and curcumin indicated synergistic inhibitory effects on cell viability, wound healing, cell migration and invasion, and primary tumor formation. To determine the selective effect of combination of “Met + Cur” on cancerous cells, very low doses of 8 anticancer drugs (cisplatin, carboplatin, oxaliplatin, epirubicin, doxorubicin, docetaxel, paclitaxel, and methotrexate) used in MTT assay were comparatively tested on AGS cancer cells and normal HDF cells for 48 and 72 hours. The results indicated that the combination of “Met + Cur” significantly increased cytotoxic effects of all anticancer drugs of AGS cells. It is while in normal HDF cells, combination of “Met + Cur” along with anticancer drugs had no effect. This can be inferred as selectively additive effect.

## Introduction

Gastric cancer is one of the fifth most common type of cancer and its current treatments fundamentally depend on surgery and chemotherapy (Courtois et al., 2019[7]). However, almost in 20-30 % of cases, high rates of local recurrence and metastasis, low survival rates, and significantly decreased life quality are observable (Farajzadeh et al., 2018[11]; Lu et al., 2019[20]). Consequently, new therapies for improved gastric cancer care with minimal side effects is necessary. Metformin (Met) and curcumin (Cur) are promising candidates for such therapeutic applications (Farajzadeh et al., 2018[11]). 

Metformin is a biguanide drug and the first-line treatment for type 2 diabetes mellitus. Its mechanism of action is through enhancing insulin sensitivity in hepatic (decreased gluconeogenesis) and peripheral (stimulation of glucose absorption in muscles and/or increased fatty acid oxidation in adipocytes) tissues (Lu et al., 2019[20]). Epidemiologic data show that metformin administration correlates with significantly decreased cancer incidence, enhanced prognosis, and lower cancer mortality in type 2 diabetes mellitus patients (DeCensi et al., 2010[8]). 

Curcumin is a polyphenolic compound available in turmeric with a long history of usage in traditional medicine. Today, curcumin is used for treating flatulence, arthritis, sprains, stomach discomfort, jaundice, skin infections, wounds, etc. (Guo et al., 2015[14]). This compound has some health-improving characteristics like anti-aging, anti-hypertensive, anti-inflammation, and anti-cancer activities. It is reported to possess antitumor effects in several cancers, including gastric cancer (Ye et al., 2019[32]). Some studies revealed anti-proliferative and anti-metastatic activities of metformin in several cancers (Suissa and Azoulay, 2014[27]). Curcumin also suppresses proliferation and induces apoptosis in cancer cell (Suissa and Azoulay, 2001[27]). 

The newest human cancer treatments use efficient combinations of therapeutic agents. A well-targeted combinatorial strategy may improve anticancer effects, or even lead to synergistic inhibitory effects on carcinogenesis. Presently, approaches mainly focus on higher efficacy of combinatorial treatments (Farajzadeh et al., 2018[11]). The effect of curcumin and metformin co-treatment has been paid a special attention during the last two years. Studies with hepatocellular carcinoma (Zhang et al., 2018[33]), breast cancer (Falah et al., 2017[10]; Farajzadeh et al., 2018[11]; McCubrey et al., 2018[21]), and prostate cancer (McCubrey et al., 2018[21]) cell lines, and oral cancer models (Siddappa et al., 2017[26]) indicate synergistic effects of metformin and curcumin co-treatment on apoptosis induction, inhibition of migration and suppressed tumor growth. 

Nevertheless, the effects of treating gastric cancer with metformin and curcumin are unknown, particularly for combinatorial treatment. Regarding the novel investigations in this field and lack of reports about effectiveness of co-treatments of gastric cancer cell lines with metformin and curcumin (in particular, its specific cytotoxic effect on cancer cell line), studies in this area seem necessary. 

The main purpose of this study was to investigate the anticancer effects of metformin and curcumin co-treatment on human AGS gastric adenocarcinoma cell line. We also aimed at examining the selective cytotoxicity of chemotherapy drugs in combination with metformin and curcumin in the cancer cell line.

## Materials and Methods

### Reagents and cell lines

Metformin (Santa Cruz Biotechnology, USA) and curcumin (Merck, Germany) were dissolved in RPMI 1640 culture medium (Bio-Idea, Iran) and DMSO (Shellmax, China), respectively. Anticancer drugs cisplatin (Mylan, USA), carboplatin (Mylan, USA), oxaliplatin (Sobhan, Iran), epirubicin (Ebewe, Austria), doxorubicin (Ebewe, Austria), docetaxel (Sanofi, France), paclitaxel (Sobhan, Iran), and methotrexate (Mylan, USA) were used. Cell proliferation kit (MTT) was provided from Roche (Switzerland). Matrigel Basement Membrane Matrix Growth Factor Reduced (Cat No. 354230) and Transwells (Cat No. 3422) were products of Corning (USA). Human AGS gastric cell line and human HDF normal cell line were purchased from the American Type Culture Collection (ATCC), were cultivated in RPMI 1640 and DMEM/F12 culture media, respectively, supplemented with heat-inactivated fetal bovine serum (FBS, Gibco, USA, 10 % v/v), 100 units/mL penicillin, and 100 μg/mL streptomycin (Bio-Idea, Iran).

### MTT assay

According to standard protocol (Kumar et al., 2018[18]), 5×10^3^ cells/well were cultivated in a 96-well plate. After 24 hours, single treatments with metformin and curcumin were performed for 24, 48, 72, and 96 hours. Half maximal inhibitory concentration (IC_50_) values were calculated with GraphPad Prism Software Version 7.01 (GraphPad Software Inc., USA). Combinatorial treatments were conducted regarding IC_50_ values for 48 (with a ratio of 1250:1 metformin to curcumin) and 72 (with a ratio of 625:1 metformin to curcumin) hours. Final concentrations of metformin and curcumin in single and combinatorial forms were tested with AGS and HDF cell lines. 

Also, eight anticancer drugs (cisplatin, carboplatin, oxaliplatin, epirubicin, doxorub-icin, docetaxel, paclitaxel, and methotrexate) were comparatively tested on two cell lines with or without combination of metformin and curcumin. 

### Synergistic quantification of combination

Synergistic effect combination of metfor-min and curcumin was determined based on Chou-Talalay method using CompuSyn Software (Chou, 2006[6]).

### Cell migration tests 

Two-dimensional motility of cells was tested using wound healing and Transwell methods (Justus et al., 2014[17]). In wound healing method, after cultivating cells in 6-well plates and reaching the proper confluency of about 70-80 %, scratches were made using sterile 100-μL pipette tips. Metformin (0.625 mM), curcumin (1 μM), and their combination were added to wells. Control wells were supplemented with vehicle. Scratches were photographed using an inverted microscope (BEL, Italy) at 0, 24, 48, and 72 hours post treatments. The scratch area was calculated by the aid of ImageJ Software Version 1.6.0. Migration capacity was also explored by Transwell method in 24-well plates. 7×10^4^ cells were suspended in 100 μL of serum-free medium and added to the upper chamber of a Transwell with single and combinatorial concentrations of metformin and curcumin. The lower chamber contained 600 μL of FBS-supplemented culture medium. After 24 hours, the cells migrating to the lower chamber were fixed with 70 % ethanol solution (Merck, Germany), stained with 0.5 % (w/v) crystal violet dye (Merck, Germany), and counted under a bright field microscope (BEL, Italy).

### Cell invasion assay

Invasive characteristics of human AGS cells were examined with conditions similar to cell migrations assay (Justus et al., 2014[17]). The only difference was that before adding cell suspension, 100 μL of Corning Matrigel Matrix was added to the upper chamber and incubated at 37 °C for 1 hour.

### Colony formation assay 

Colony formation assay is a method for determining tumorigenic potential of cells. According to definitions, colonies must include at least 50 cells (Franken et al., 2006[12]; Rajendran and Jain, 2018[25]). A total number of 400 cells were seeded and simultaneously treated with mentioned doses of metformin, curcumin, and their combination in each 3.5-cm Petri dish. After fixation with methanol-acetone (1:1) and staining with crystal violet on day 12, colonies were counted using a colony counter.

### Statistical analysis

All experiments were done in three biological independent experiments. Data is expressed as the mean ± standard deviation (SD). Statistical analysis was performed using correlation analysis, one-way analysis of variance (ANOVA) and Duncan's post-hoc test in SPSS 16.0 Software (SPSS, Inc., USA) with a significance level less than 0.05 *(p*<0.05).

## Results

### Combination of metformin and curcumin synergistically inhibits AGS cell viability

**Figure 1:**
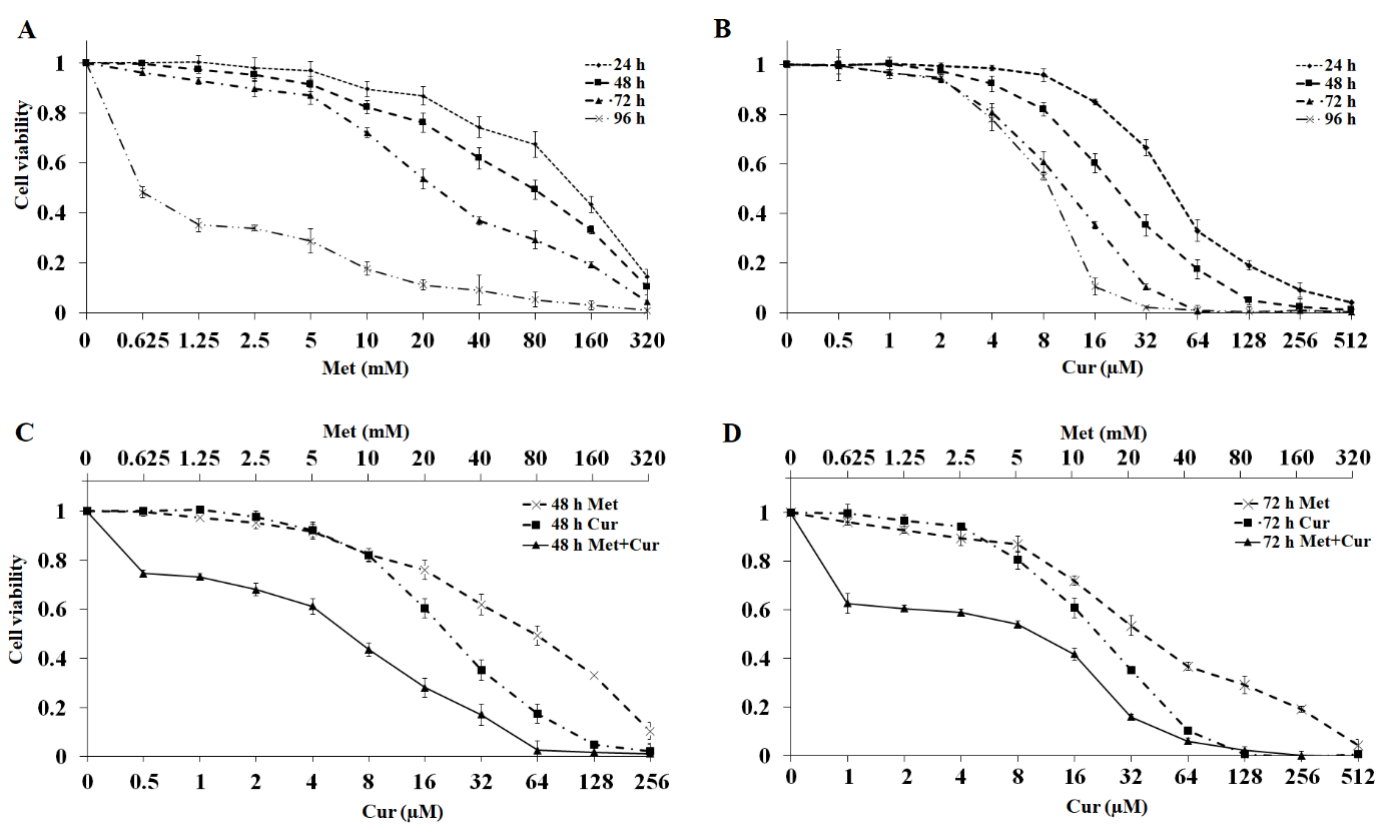
Combination of metformin (Met) and curcumin (Cur) synergistically inhibits viability of AGS cell line. A and B: AGS cell viability (mean ± SD) after treatment with metformin and curcumin for 24, 48, 72, and 96 hours. C and D: AGS cell viability (mean ± SD) for combinatorial treatment with metformin and curcumin (1250:1 for 48 hours and 625:1 for 72 hours). Doses of metformin in the top row and curcumin in the bottom row of the graph are presented, and both should be considered for combination charts. Each experiment was performed independently in triplicate for each data point.

Treatment of AGS cells with metformin and curcumin for 24 to 96 hours and calculation of cell viability values indicated significant dose- and time-dependent effects (*p*<0.001) for single treatments (Figure 1A, 1B(Fig. 1)). In combinatorial treatments, Met to Cur ratios of 1250:1 for 48-hour treatments and 625:1 for 72-hour treatments were specified based on IC_50_ values in single dose-response curves. MTT assay for combination was conducted with a concentration gradient of selected ratios. To analyze the exact nature of interactions between metformin and curcumin in a combination, synergistic effect was determined through Chou-Talalay method by the aid of CompuSyn Software (Table 1(Tab. 1)). 

In this method, combination index (CI) graphs showed that combinatorial treatment significantly decreased cell viability and had a time-dependent synergistic growth inhibitory effect. Although single doses of metformin and curcumin have insignificant cytotoxic effects on AGS cells in both 48 and 72 hours (viability > 97 %) (Figure 1C, 1D(Fig. 1)), their combination shows a significant cytotoxicity (*p*<0.001). Based on results of synergistic effect examination, single and combinatorial dosages of 0.625 mM for metformin and 1 μM for curcumin were selected for further tests.

### Combination of metformin and curcumin inhibits metastatic potential of AGS cells

**Figure 2:**
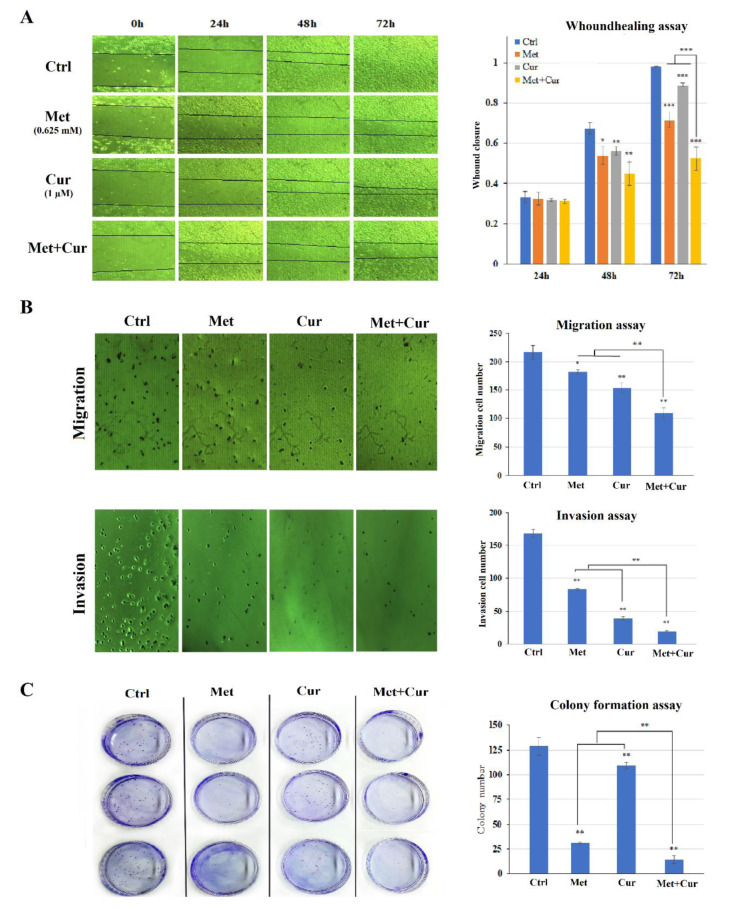
Combination of metformin (Met) and curcumin (Cur) synergistically inhibits metastatic potential in AGS cell line compared to the control group (Ctrl). (A) Migration of AGS cells treated with metformin (0.625 mM), curcumin (1 μM), and combination (0.625 mM metformin + 1 μM curcumin) for 24, 48, and 72 hours was examined through wound healing assay by the aid of ImageJ software. (B) Cell migration and invasion were inspected via transwell method (without matrigel and with matrigel coating, respectively) 24 hours after treatment with metformin (0.625 mM), curcumin (1 μM), and their combination. (C) Observation of tumorigenic characteristics of AGS cells treated with metformin (0.625 mM), curcumin (1 μM), and their combination via colony formation assay during 12 days. Each column in graphs represents mean ± SD for three independent experiments and compared with untreated control determined by the ANOVA test. *, ** and *** show significant differences at p<0.05, p<0.01 and p<0.001, respectively.

Metformin and/or curcumin potential of inhibiting migration, invasion, and tumorigenesis were assessed. Wound healing was rapid in control group, so that the scratch closed after 72 hours, while in groups with single treatments, this process was significantly inhibited. The combination of metformin and curcumin was more effective in a time- and dose-dependent manner (Figure 2A)(Fig. 2). 

Similar results were obtained in Transwell experiment. As shown in Figure 2B(Fig. 2), in comparison to single treatments, combination of metformin and curcumin significantly blocks cell migration. Furthermore, invasion rate of AGS cells was inspected. Statistical analysis indicated significant inhibitory effects of single and combinatorial treatments on cell invasion rates (Figure 2B(Fig. 2)). 

Colony formation assay was exploited to study the inhibitory effect of metformin and/or curcumin on tumorigenesis. Counting colonies with more than 50 cells showed that treating with concentration of metformin and curcumin could significantly inhibit colony formation. The combination had a higher inhibitory effect than single treatments (Figure 2C(Fig. 2)).

### Combination of metformin and curcumin enhances cytotoxic effects of chemotherapeutic drugs on AGS cells

**Figure 3:**
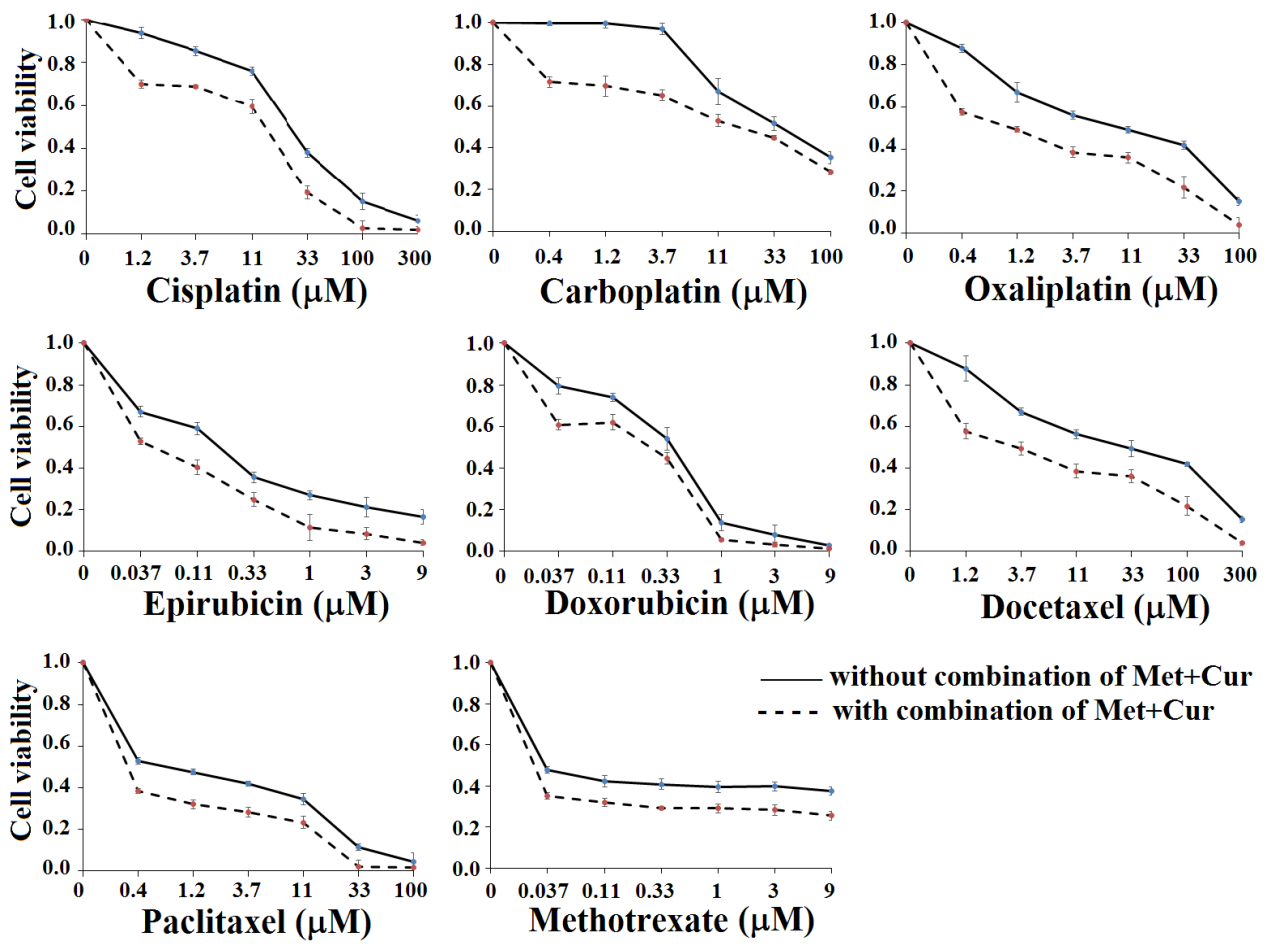
AGS cell line viability (mean ± SD) after treatment with 3-fold serial dilutions of anticancer drugs with or without combination of metformin (Met, 0.625 mM) + curcumin (Cur, 0.5 µM) for 48 hours. All experiments were carried out independently in triplicate. “Met + Cur” significantly increases the cytotoxic effects of anticancer drugs.

MTT test results on AGS cells with anti-cancer drugs cisplatin, carboplatin, oxaliplatin, epirubicin, doxorubicin, docetaxel, paclitaxel, and methotrexate in single serial dilution form with and without fixed concentrations of metformin and curcumin combination for 48 hours (0.625 mM Met + 0.5 μM Cur) were considered. Statistical analysis showed that the cytotoxic effect was enhanced in the presence of “Met + Cur” combination with each drug in a dose-dependent manner (*p*<0.05) (Figure 3(Fig. 3)). Similar results were observed from repeated experiments for 72 hours (Supplementary Figure 1).

**Figure 4:**
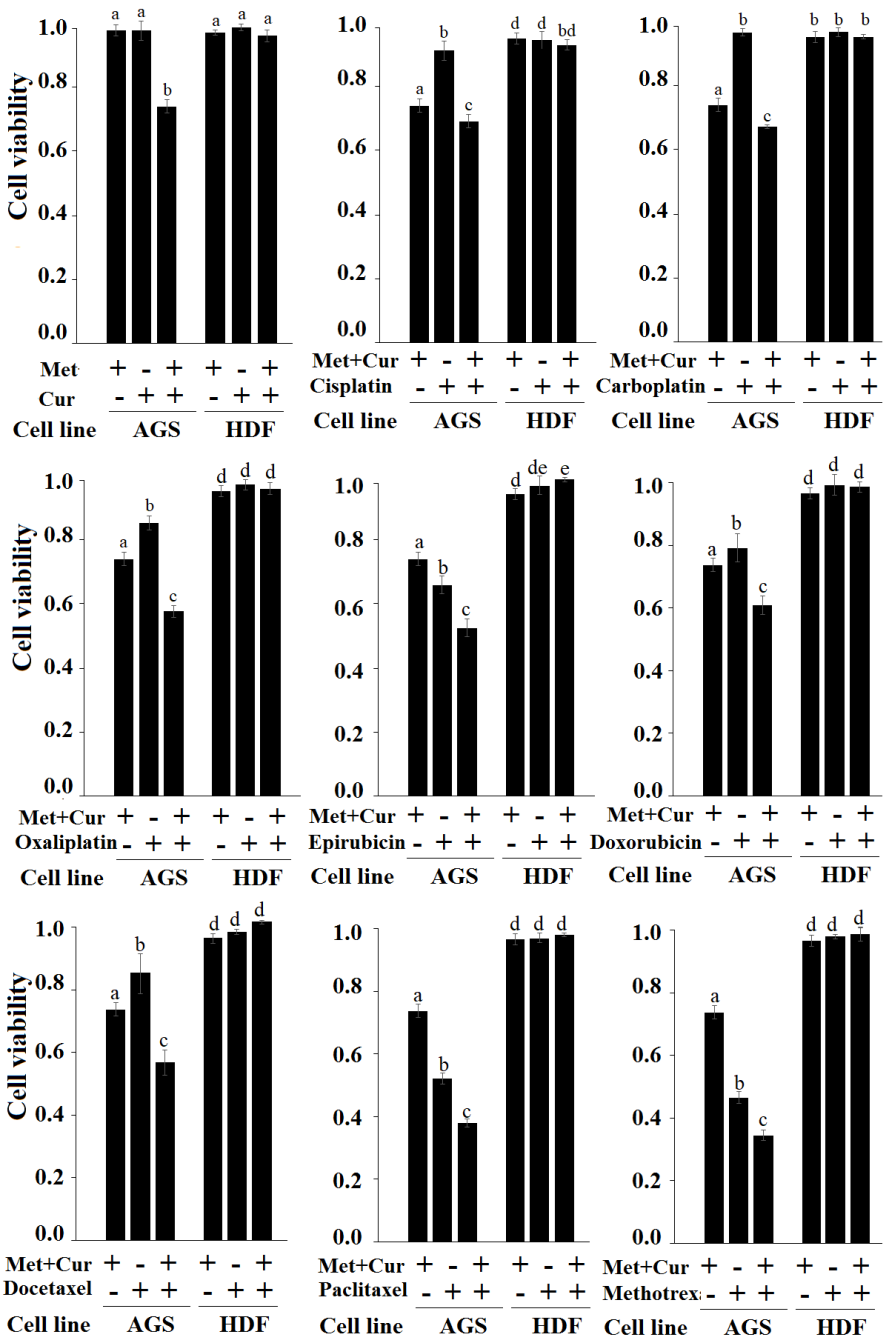
Treatment of cancerous AGS and normal HDF cells with metformin (Met), curcumin (Cur), “Met + Cur”, and anticancer drugs (with and without combination of “Met + Cur”) for 48 hours. Met, Cur, cisplatin, carboplatin, oxaliplatin, epirubicin, doxorubicin, docetaxel, paclitaxel, and methotrexate were used at final concentrations of 0.625 mM, 0.5µM, 1.2 µM, 0.4 µM, 0.4 µM, 37 nM, 37 nM, 1.2 µM, 0.4 mM, and 37 nM, respectively. The results are the means of three independent experiments. Statistical analysis was performed using one-way ANOVA with Duncan's post-hoc test. Analysis indicates an increase in the specific cytotoxicity of anticancer drugs in the presence of “Met + Cur”. In each panel, a similar alphabet does not imply statistical significance.

To determine the selective effect of combination of “Met + Cur” on cancerous cells, eight anticancer drugs, cisplatin, carboplatin, oxaliplatin, epirubicin, doxorubicin, docetaxel, paclitaxel, and methotrexate at final concentrations of 1.2 μM, 0.4 μM, 0.4 μM, 37 nM, 37 nM, 1.2 μM, 0.4 μM, and 37 nM, respectively, were comparatively tested on cancerous AGS cells and normal HDF cells for 48 hours. The results indicated that the combination of “Met + Cur” significantly increased cytotoxic effects of all anticancer drugs of AGS cell line, so that cytotoxicity of triple combination of “Met + Cur” and anticancer drug had a significant difference with single drug or “Met + Cur” alone (*p*<0.01). In normal HDF cells, the combination of "Met + Cur" together with anticancer drugs had no effect. This can be inferred as selectively additive effect. As previously mentioned, the single doses of metformin and curcumin at 48 hours did not significantly affect neither AGS nor HDF, but the combination of these two substances showed a significant cytotoxicity effect on AGS cells. This effect was selective and was not observed in normal HDF cells (Figure 4(Fig. 4)). 

Similar results were observed from repeated experiments for 72 hours (Supplementary Figure 2).

See also the Supplementary Data.

## Discussion

Recently, epidemiologic, preclinical, and clinical evidence have shown that pharmacologic inhibition of single targets only yields proper responses in a few cancer patients. This could weaken the efficacy of drugs (Wu et al., 2015[30]). In order to improve survival rate and efficiency of treatments, therapeutic strategies based on the combination of two agents have been interesting (Gupta et al., 2011[15]). However, the effectiveness of treating gastric cancer with metformin and curcumin are unclear, particularly in their combination. The present study aimed at the assessment of anticancer effects of co-treatments with metformin and curcumin on a human gastric cancer cell line and HDF normal cell line.

Results of the present study showed that combinatorial concentrations of metformin and curcumin synergistically inhibited AGS cancerous cell line viability (CI<1) and hindered migration, invasion, and colony formation, while this synergistic effect was not apparent for HDF normal cell line. In the present work, it was proven that in contrast to the normal HDF cells, “Met + Cur” combination could significantly increase selective cytotoxic effects of chemotherapeutic agents on AGS cancerous cell line. Despite recent studies indicate that either metformin (Iliopoulos et al., 2011[16]; Peng et al., 2017[23]; Wu, 2017[31]) or curcumin (McCubrey et al., 2018[21]; Tan and Norhaizan, 2019[28]) can increase efficiency of chemotherapeutics, combination of metformin and curcumin with these drugs and their selective effects on cancer cells have not been investigated up to now. Noting two unresolved obstacles of increasing resistance and toxicity of chemotherapeutic agents (Amable, 2016[2]), combinatorial treatments with low-complication compounds can attenuate toxicity of anticancer drugs and improve their efficiency. 

In addition to epidemiological studies establishing dose- and time-dependent, preventive and protective roles of metformin in lowering cancer incidence and mortality in type 2 diabetes mellitus patients (Evans et al., 2005[9]). Metformin can also induce apoptosis, impede proliferation, tumorigenesis, metastasis and angiogenesis in a dose-dependent manner in several cancer cell types (Evans et al., 2005[9]; Buzzai et al., 2007[4]; Poli et al., 2016[24]; Falah et al., 2017[10]). Population-based investigations have also emphasized on potential of curcumin as an anticancer agent (Mohandas and Desai, 1999[22]). Nevertheless, there are not any reports about the effects of co-treatment with metformin and curcumin on gastric cancer cell lines. The concentrations of metformin and curcumin used in our study are noticeably lower than some similar studies, so that anticancer effects of metformin have been reported in the range of 2.5-50 mM (Chen et al., 2015[5]; Poli et al., 2016[24]; Valaee et al., 2017[29]) and for curcumin, in the range of 10-110 μM (Falah et al., 2017[10]; Liu et al., 2018[19]; Abdelsamia et al., 2019[1]). Although these low concentrations do not have cytotoxic effects in single treatments, they exert notable anti-cancer effects in combination and these effects are comparable with the results of similar studies.

In cancer, combination therapy has been shown to be more effective than monotherapy. Monotherapy non-selectively targets rapidly growing cells, and chemotherapy results in high toxicity and immunosuppression. Because combination therapy works synergistically or additively, lower doses of individual drugs are prescribed, potentially reducing drug resistance problems in tumor cells and the drug toxicity for healthy cells (Bayat Mokhtari et al., 2017[3]; Ghosh et al., 2018[13]). The usage of very low concentrations, synergistic anticancer effect of combinatorial treatments, and especially increased toxicity of chemotherapeutic drugs in a cancerous cell line versus minimum adverse effects on a normal cell line are among highlights of our investigation. Although in accordance with the objectives of our study metformin and curcumin have significant effects on the AGS gastric cancer cell line, in vivo studies could seemingly shed more light on this field.

In general, these findings show that combination of metformin and curcumin might be a potential candidate for further investigations in the field of gastric cancer treatment. 

## Declaration

### Acknowledgments

This study was supported by Shiraz University (97GCU3M1740).

### Conflict of interest 

The authors have no conflicts of interest.

## Supplementary Material

Supplementary information

Supplementary data

## Figures and Tables

**Table 1:**
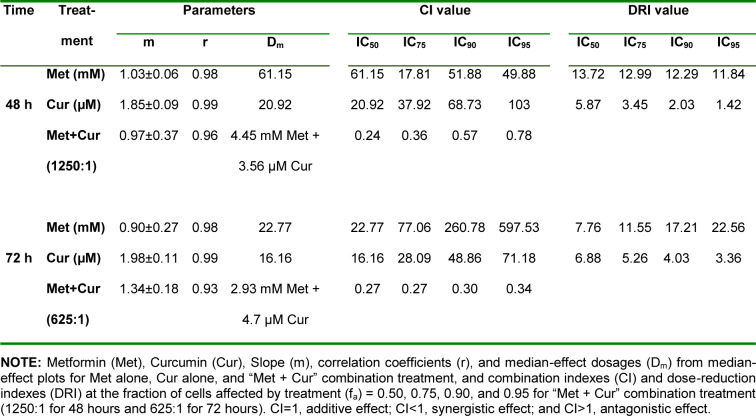
Metformin (Met) and curcumin (Cur) combination synergistically inhibits cell viability in AGS cells
