# Supplementary material for: Synergistic effects of metformin and curcumin on cytotoxicity of chemotherapy drugs using a gastric cancer cell line model
Source: EXCLI J. 2021 Oct 11;20:1488–98. doi: 10.17179/excli2021-4091 (PMC8678059; doi:10.17179/excli2021-4091)
Supplement: Supplementary information [file EXCLI-20-1488-s-001.pdf]

EXCLI Journal 2021;20:1488-1498 – ISSN 1611-2156
Received: July 13, 2021, accepted: September 29, 2021, published: October 11, 2021

Supplementary information to:

Original article:

SYNERGISTIC EFFECTS OF METFORMIN AND CURCUMIN ON CYTOTOXICITY OF CHEMOTHERAPY DRUGS USING A GASTRIC CANCER CELL LINE MODEL

Ehsan Zarei, Youssof Sefidi-Heris, Iraj Saadat*

Department of Biology, College of Sciences, Shiraz University, Shiraz, Iran

* **Corresponding author:** Iraj Saadat, Department of Biology, College of Sciences, Shiraz University, Shiraz 71467-13565, Iran. Tel: +98 71 36137435, E-mail: isaadat@shirazu.ac.ir

http://dx.doi.org/10.17179/excli2021-4091

This is an Open Access article distributed under the terms of the Creative Commons Attribution License (http://creativecommons.org/licenses/by/4.0/).

EXCLI Journal 2021;20:1488-1498 – ISSN 1611-2156
Received: July 13, 2021, accepted: September 29, 2021, published: October 11, 2021

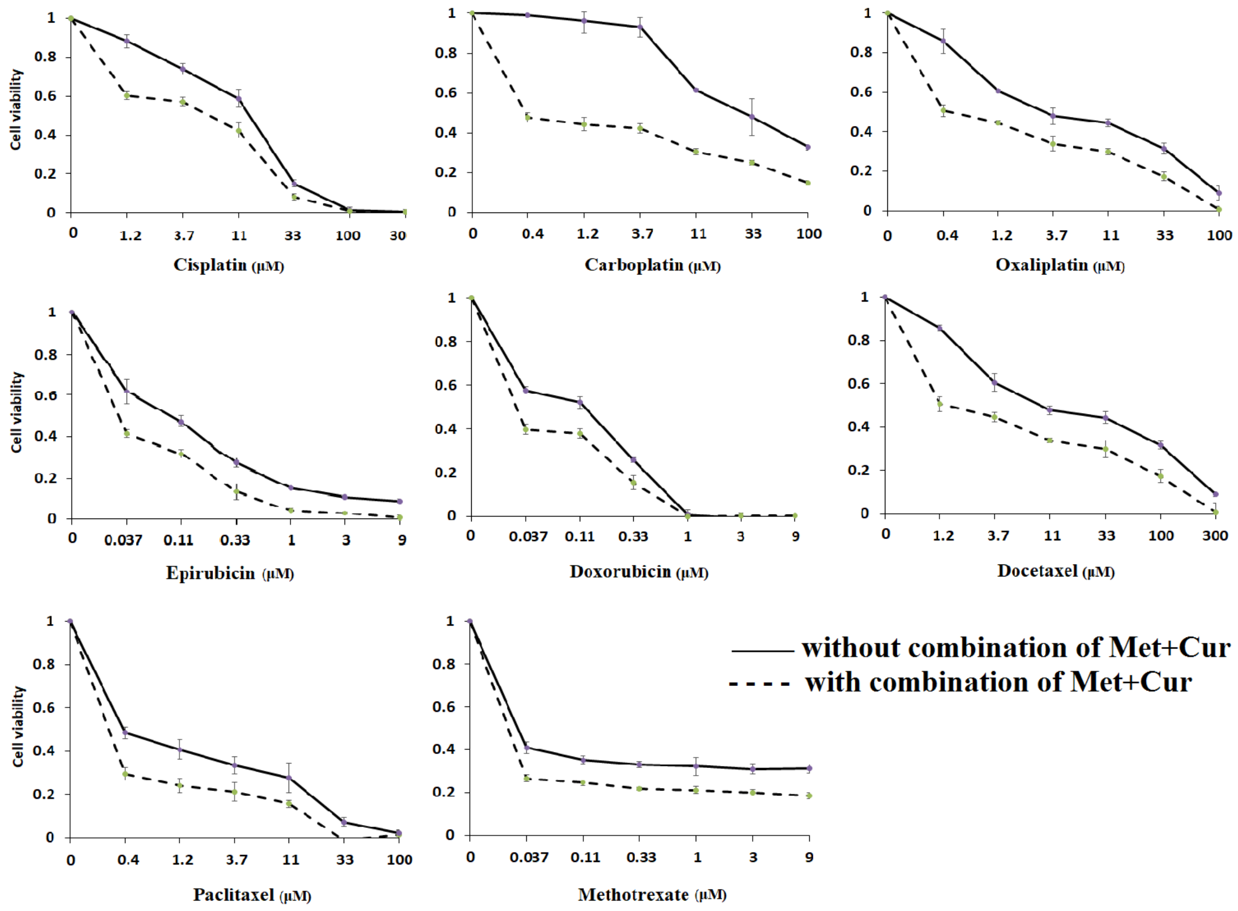

Supplementary Figure 1: AGS cell line viability (mean ± SD) after treatment with 3-fold serial dilutions of anticancer drugs with or without combination of metformin (Met) 0.625 mM + curcumin (Cur) 1 µM for 72 hours. Met+Cur significantly increases the cytotoxic effects of anticancer drugs. All experiments were carried out independently in triplicate.

Received: July 13, 2021, accepted: September 29, 2021, published: October 11, 2021

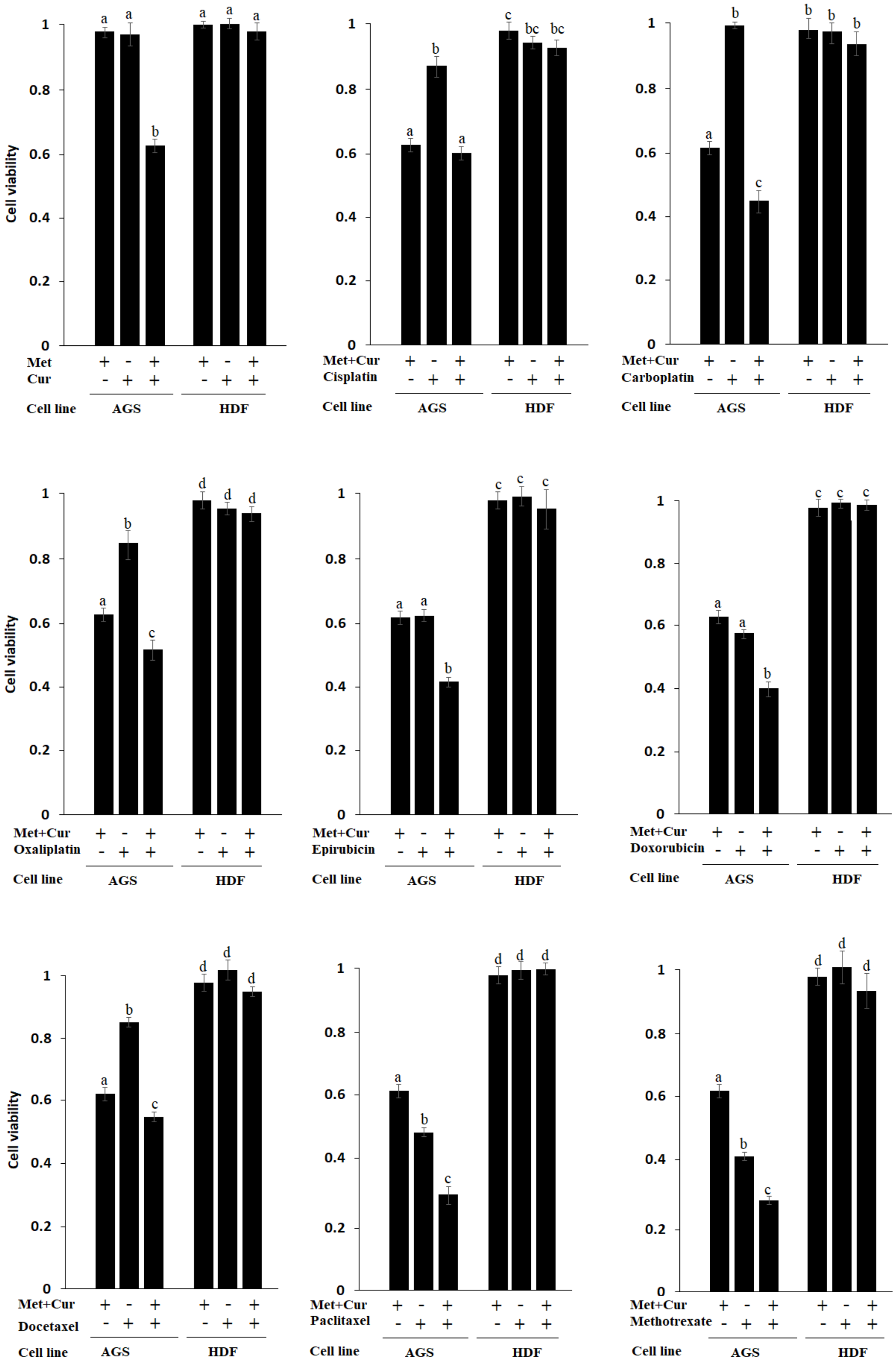

Supplementary Figure 2: Treatment of cancerous AGS and normal HDF cells with metformin (Met), curcumin (Cur), Met+Cur, and anticancer drugs (with and without combination of Met+Cur) for 72 hours. Met, Cur, cisplatin, carboplatin, oxaliplatin, epirubicin, doxorubicin, docetaxel, paclitaxel, and methotrexate were used at final concentrations of 0.625 mM, 1 µM, 1.2 µM, 0.4 µM, 0.4 µM, 37 nM, 37 nM, 1.2 µM, 0.4 µM, and 37 nM, respectively. Statistical analysis was performed using one-way ANOVA with Duncan's post-hoc test. Analysis indicates an increase in the specific cytotoxicity of anticancer drugs in the presence of Met+Cur. All experiments were done independently in triplicate. In each panel, a similar alphabet does not imply statistical significance.
