# Supplementary material for: Synergistic effects of metformin and curcumin on cytotoxicity of chemotherapy drugs using a gastric cancer cell line model
Source: EXCLI J. 2021 Oct 11;20:1488–98. doi: 10.17179/excli2021-4091 (PMC8678059; doi:10.17179/excli2021-4091)
Supplement: Supplementary data [file EXCLI-20-1488-s-002.pdf]

EXCLI Journal 2021;20:1488-1498 – ISSN 1611-2156
Received: July 13, 2021, accepted: September 29, 2021, published: October 11, 2021

Supplementary data to:

Original article:

SYNERGISTIC EFFECTS OF METFORMIN AND CURCUMIN ON CYTOTOXICITY OF CHEMOTHERAPY DRUGS USING A GASTRIC CANCER CELL LINE MODEL

Ehsan Zarei, Youssof Sefidi-Heris, Iraj Saadat*

Department of Biology, College of Sciences, Shiraz University, Shiraz, Iran

* **Corresponding author:** Iraj Saadat, Department of Biology, College of Sciences, Shiraz University, Shiraz 71467-13565, Iran. Tel: +98 71 36137435, E-mail: isaadat@shirazu.ac.ir

http://dx.doi.org/10.17179/excli2021-4091

This is an Open Access article distributed under the terms of the Creative Commons Attribution License (http://creativecommons.org/licenses/by/4.0/).

EXCLI Journal 2021;20:1488-1498 – ISSN 1611-2156
Received: July 13, 2021, accepted: September 29, 2021, published: October 11, 2021

Raw data of Figure 1A:
Cell viability of AGS cells exposed to metformin for 24, 48, 72, 96 hours. In each independent biological replication, cell viability was normalized by untreated control cell culture.

Time	Replicate	Metformin concentration (mM)										
		**0***	**0.625**	**1.25**	**2.5**	**5**	**10**	**20**	**40**	**80**	**160**	**320**
**24 h**	**1**	1	0.994	0.977	0.969	1.010	0.863	0.883	0.779	0.713	0.414	0.107
	**2**	1	1.011	1.005	1.025	0.941	0.924	0.893	0.750	0.695	0.469	0.162
	**3**	1	0.997	1.027	0.945	0.959	0.902	0.826	0.697	0.615	0.414	0.160
**48 h**	**1**	1	1.014	0.960	0.942	0.895	0.845	0.717	0.667	0.454	0.337	0.115
	**2**	1	0.988	0.986	0.935	0.951	0.794	0.772	0.585	0.490	0.342	0.131
	**3**	1	0.986	0.971	0.981	0.900	0.828	0.792	0.604	0.532	0.312	0.064
**72 h**	**1**	1	0.958	0.931	0.927	0.900	0.733	0.576	0.381	0.324	0.195	0.068
	**2**	1	0.976	0.915	0.866	0.833	0.729	0.536	0.373	0.296	0.200	0.045
	**3**	1	0.987	0.940	0.892	0.876	0.698	0.495	0.349	0.255	0.178	0.015
**96 h**	**1**	1	0.507	0.321	0.347	0.314	0.200	0.129	0.129	0.044	0.044	0.044
	**2**	1	0.466	0.371	0.341	0.316	0.178	0.113	0.118	0.083	0.011	0.000
	3	1	0.470	0.358	0.326	0.230	0.147	0.088	0.023	0.026	0.032	0.014

*: Factor values examined were normalized to a dose of zero (control, untreated) and considered as one factor.

EXCLI Journal 2021;20:1488-1498 – ISSN 1611-2156
Received: July 13, 2021, accepted: September 29, 2021, published: October 11, 2021

Raw data of Figure 1B:
Cell viability of AGS cells exposed to curcumin for 24, 48, 72, and 96 hours. In each independent biological replication, cell viability was normalized by untreated control cell culture.

Time	Replicate	Curcumin concentration (µM)											
		0	0.5	1.0	2.0	4.0	8.0	16	32	64	128	256	512
24 h	1	1	0.938	1.014	0.984	0.984	0.985	0.861	0.629	0.282	0.212	0.120	0.064
	2	1	1.016	1.001	0.994	0.999	0.957	0.839	0.675	0.367	0.174	0.071	0.043
	3	1	1.061	0.997	1.006	0.978	0.933	0.844	0.693	0.340	0.192	0.098	0.028
48 h	1	1	0.950	0.988	0.941	0.879	0.782	0.558	0.311	0.145	0.042	0.025	0.022
	2	1	1.020	0.995	0.965	0.913	0.845	0.598	0.342	0.156	0.051	0.019	0.019
	3	1	1.028	1.011	1.015	0.979	0.838	0.653	0.403	0.219	0.051	0.019	0.010
72 h	1	1	0.962	0.941	0.924	0.773	0.626	0.338	0.109	0.018	0.005	0.023	0.003
	2	1	1.033	0.988	0.955	0.799	0.639	0.367	0.111	0.004	0.005	0.004	0.002
	3	1	0.994	0.974	0.949	0.847	0.562	0.352	0.093	0.004	0.011	0.002	0.008
96 h	1	1	1.033	0.988	0.963	0.813	0.559	0.149	0.024	0.023	0.018	0.023	0.001
	2	1	0.994	0.974	0.936	0.748	0.539	0.060	0.018	0.004	0.009	0.004	0.001
	3	1	0.996	0.968	0.949	0.781	0.549	0.104	0.021	0.009	0.004	0.009	0.001

EXCLI Journal 2021;20:1488-1498 – ISSN 1611-2156
Received: July 13, 2021, accepted: September 29, 2021, published: October 11, 2021

Raw data of Figure 1C:
Cell viability of AGS cells exposed to combination of metformin and curcumin for 48 hours. In each independent biological replication, cell viability was normalized by untreated control cell culture.

Replicate	Metformin (mM) + Curcumin (µM) concentrations										
	**0**	**0.625+0.5**	**1.25+1**	**2.5+2**	**5+4**	**10+8**	**20+16**	**40+32**	**80+64**	**160+128**	**320+256**
**1**	1	0.732	0.720	0.657	0.632	0.456	0.310	0.159	0.056	0.010	0.017
**2**	1	0.738	0.739	0.714	0.584	0.439	0.242	0.214	0.007	0.070	0.006
**3**	1	0.766	0.738	0.684	0.622	0.414	0.289	0.140	0.044	0.024	0.061

Raw data of Figure 1D:
Cell viability of AGS cells exposed to combination of metformin and curcumin for 72 hours. In each independent biological replication, cell viability was normalized by untreated control cell culture.

Replicate	Metformin (mM) + Curcumin (µM) concentrations										
	**0**	**0.625+1**	**1.25+2**	**2.5+4**	**5+8**	**10+16**	**20+32**	**40+64**	**80+128**	**160+256**	**320+512**
**1**	1	0.593	0.612	0.600	0.563	0.449	0.171	0.075	0.029	0.014	0.011
**2**	1	0.656	0.618	0.597	0.547	0.379	0.163	0.062	0.036	0.008	0.005
**3**	1	0.600	0.590	0.572	0.512	0.427	0.148	0.047	0.006	0.004	0.001

EXCLI Journal 2021;20:1488-1498 – ISSN 1611-2156
Received: July 13, 2021, accepted: September 29, 2021, published: October 11, 2021

Raw data of Figure 2A:
Migration of AGS cells treated with metformin, curcumin and their combination for 24, 48, and 72 hours in three independent replications was examined through wound healing assay by the aid of ImageJ software.

Treatment	Replicate	Time											
		0 h			24 h			48 h			72 h		
		Scratch area (Mega pixels)	Scratch area ratio*	Wound closure**	Scratch area (Mega pixels)	Scratch area ratio*	Wound closure**	Scratch area (Mega pixels)	Scratch area ratio*	Wound closure**	Scratch area (Mega pixels)	Scratch area ratio*	Wound closure**
**Control**	**1**	2.069	1	0	1.429	0.690	0.310	0.662	0.320	0.680	0.035	0.017	0.983
	**2**	2.069	1	0	1.316	0.636	0.364	0.621	0.300	0.700	0.033	0.016	0.984
	**3**	2.097	1	0	1.419	0.677	0.323	0.753	0.359	0.641	0.041	0.020	0.980
**0.6 mM Metformin**	**1**	1.915	1	0	1.314	0.686	0.314	0.971	0.507	0.493	0.560	0.292	0.708
	**2**	2.112	1	0	1.350	0.639	0.361	0.883	0.418	0.582	0.524	0.248	0.752
	**3**	2.013	1	0	1.409	0.700	0.300	0.927	0.460	0.540	0.643	0.319	0.681
**1 µM Curcumin**	**1**	2.093	1	0	1.419	0.678	0.322	0.965	0.461	0.539	0.215	0.103	0.897
	**2**	2.208	1	0	1.496	0.677	0.323	0.934	0.423	0.577	0.264	0.120	0.880
	**3**	2.092	1	0	1.446	0.691	0.309	0.901	0.431	0.569	0.229	0.109	0.891
**0.6 mM Metformin + 1 µM Curcumin**	**1**	2.212	1	0	1.510	0.683	0.317	1.195	0.540	0.460	0.952	0.430	0.570
	**2**	2.251	1	0	1.529	0.679	0.321	1.123	0.499	0.501	1.037	0.461	0.539
	**3**	2.059	1	0	1.444	0.702	0.298	1.258	0.611	0.389	1.109	0.539	0.461

* Scratch area ratio in each treatment group = Scratch area at any time ÷ Scratch area at 0 h.
** Wound closure= 1- Scratch area ratio

EXCLI Journal 2021;20:1488-1498 – ISSN 1611-2156
Received: July 13, 2021, accepted: September 29, 2021, published: October 11, 2021

Raw data of Figure 2B and 2C:
Cell migration and invasion were inspected via transwell method (without matrigel and with matrigel coating, respectively) 24 hours after treatment with metformin, curcumin and their combination. The colony formation of AGS cells treated with metformin, curcumin and their combination after 12 days were evaluated by counting the number of colonies.

Treatment	Replicate	Migrated cell	Invaded cell	Formed colony
**Control**	**1**	230	170	128
	**2**	206	164	127
	**3**	216	171	131
**0.6 mM Metformin**	**1**	186	85	30
	**2**	179	78	33
	**3**	181	86	31
**1 µM Curcumin**	**1**	162	38	111
	**2**	146	41	108
	**3**	153	38	107
**0.6 mM Met-formin + 1 µM Curcumin**	**1**	119	21	14
	**2**	102	18	16
	**3**	108	19	13

EXCLI Journal 2021;20:1488-1498 – ISSN 1611-2156
Received: July 13, 2021, accepted: September 29, 2021, published: October 11, 2021

Raw data of Figure 3: Viability of AGS cells after treatment with serial dilutions of anticancer drugs with or without combination of metformin and curcumin for 48 hours. All experiments were carried out independently in triplicate. In each independent biological replication, cell viability was normalized by untreated control cell culture.

Treatment	Replicate	Cisplatin concentration (µM)						
		**0**	**1.2**	**3.7**	**11**	**33**	**100**	**300**
**Cisplatin**	**1**	1	0.905	0.830	0.740	0.403	0.111	0.085
	**2**	1	0.955	0.874	0.762	0.365	0.186	0.068
	**3**	1	0.946	0.852	0.777	0.369	0.151	0.032
**Cisplatin + (0.6 mM Metformin + 0.5 µM Curcumin)**	**1**	1	0.654	0.682	0.568	0.180	0.001	0.004
	**2**	1	0.695	0.690	0.585	0.172	0.043	0.011
	**3**	1	0.678	0.696	0.631	0.225	0.059	0.049

Treatment	Replicate	Carboplatin concentration (µM)						
		**0**	**0.4**	**1.2**	**3.7**	**11**	**33**	**100**
**Carboplatin**	**1**	1	0.985	1.020	0.959	0.642	0.527	0.372
	**2**	1	1.000	0.995	1.002	0.627	0.480	0.365
	**3**	1	1.010	0.973	0.953	0.741	0.542	0.319
**Carboplatin + (0.6 mM Metformin + 0.5 µM Curcumin)**	**1**	1	0.745	0.747	0.652	0.561	0.447	0.292
	**2**	1	0.699	0.650	0.677	0.525	0.459	0.285
	**3**	1	0.703	0.689	0.626	0.502	0.438	0.270

Treatment	Replicate	Oxaliplatin concentration (µM)						
		**0**	**0.4**	**1.2**	**3.7**	**11**	**33**	**100**
**Oxaliplatin**	**1**	1	0.856	0.722	0.573	0.506	0.431	0.171
	**2**	1	0.894	0.658	0.574	0.493	0.395	0.154
	**3**	1	0.885	0.631	0.539	0.475	0.427	0.133
**Oxaliplatin + (0.6 mM Metformin + 0.5 µM Curcumin)**	**1**	1	0.570	0.509	0.388	0.385	0.270	0.031
	**2**	1	0.565	0.489	0.410	0.357	0.171	0.008
	**3**	1	0.596	0.480	0.357	0.332	0.209	0.105

EXCLI Journal 2021;20:1488-1498 – ISSN 1611-2156
Received: July 13, 2021, accepted: September 29, 2021, published: October 11, 2021

Raw data of Figure 3 (cont.)

Treatment	Replicate	Epirubicin concentration (µM)						
		**0**	**0.03**	**0.11**	**0.33**	**1**	**3**	**9**
**Epirubicin**	**1**	1	0.687	0.608	0.331	0.290	0.263	0.200
	**2**	1	0.631	0.604	0.383	0.268	0.169	0.129
	**3**	1	0.691	0.557	0.349	0.248	0.202	0.167
**Epirubicin + (0.6 mM Metformin + 0.5 µM Curcumin)**	**1**	1	0.512	0.419	0.217	0.173	0.114	0.026
	**2**	1	0.532	0.429	0.242	0.117	0.053	0.043
	**3**	1	0.543	0.364	0.283	0.047	0.084	0.052

Treatment	Replicate	Doxorubicin concentration (µM)						
		**0**	**0.03**	**0.11**	**0.33**	**1**	**3**	**9**
**Doxorubicin**	**1**	1	0.837	0.719	0.593	0.099	0.132	0.024
	**2**	1	0.786	0.751	0.486	0.131	0.061	0.028
	**3**	1	0.759	0.749	0.540	0.179	0.039	0.025
**Doxorubicin + (0.6 mM Metformin + 0.5 µM Curcumin)**	**1**	1	0.604	0.656	0.474	0.065	0.024	0.015
	**2**	1	0.583	0.622	0.446	0.052	0.027	0.008
	**3**	1	0.635	0.580	0.417	0.049	0.045	0.007

Treatment	Replicate	Docetaxel concentration (µM)						
		**0**	**1.2**	**3.7**	**11**	**33**	**100**	**300**
**Docetaxel**	**1**	1	0.871	0.709	0.594	0.534	0.426	0.123
	**2**	1	0.937	0.677	0.563	0.428	0.399	0.172
	**3**	1	0.826	0.689	0.539	0.499	0.408	0.089
**Docetaxel + (0.6 mM Metformin + 0.5 µM Curcumin)**	**1**	1	0.509	0.530	0.435	0.402	0.205	0.105
	**2**	1	0.552	0.470	0.372	0.335	0.263	0.031
	**3**	1	0.685	0.495	0.367	0.308	0.187	0.013

EXCLI Journal 2021;20:1488-1498 – ISSN 1611-2156
Received: July 13, 2021, accepted: September 29, 2021, published: October 11, 2021

Raw data of Figure 3 (cont.)

Treatment	Replicate	Palitaxel concentration (µM)						
		**0**	**0.4**	**1.2**	**3.7**	**11**	**33**	**100**
**Palitaxel**	**1**	1	0.539	0.473	0.425	0.334	0.125	0.087
	**2**	1	0.511	0.490	0.428	0.324	0.120	0.035
	**3**	1	0.538	0.463	0.404	0.374	0.096	0.002
**Palitaxel + (0.6 mM Metformin + 0.5 µM Curcumin)**	**1**	1	0.393	0.319	0.307	0.261	0.049	0.001
	**2**	1	0.369	0.341	0.279	0.204	0.008	0.043
	**3**	1	0.384	0.296	0.262	0.232	0.006	0.003

Treatment	Replicate	Methotrexate concentration (µM)						
		**0**	**0.03**	**0.11**	**0.33**	**1**	**3**	**9**
**Methotrexate**	**1**	1	0.463	0.447	0.446	0.424	0.383	0.368
	**2**	1	0.474	0.427	0.382	0.393	0.388	0.358
	**3**	1	0.496	0.395	0.398	0.367	0.421	0.397
**Methotrexate + (0.6 mM Metformin + 0.5 µM Curcumin)**	**1**	1	0.338	0.300	0.288	0.313	0.307	0.278
	**2**	1	0.367	0.320	0.299	0.288	0.286	0.234
	**3**	1	0.346	0.338	0.284	0.269	0.253	0.253

EXCLI Journal 2021;20:1488-1498 – ISSN 1611-2156
Received: July 13, 2021, accepted: September 29, 2021, published: October 11, 2021

Raw data of Figure 4:
Cell viability of AGS and HDF cells treated with anticancer drugs alone and in presence of "Metformin + Curcumin" for 48 hours

Treatments	AGS cells			HDF cells		
	Replicate 1	Replicate 2	Replicate 3	Replicate 1	Replicate 2	Replicate 3
**0.6 mM Metformin**	1.014	0.988	0.986	0.983	0.998	0.992
**0.5 µM Curcumin**	0.950	1.020	1.028	1.004	1.014	0.997
**Metformin + Curcumin***	0.732	0.738	0.766	0.994	0.965	0.988
**1.2 µM Cisplatin**	0.905	0.955	0.946	1.005	0.971	0.956
**1.2 µM Cisplatin + Metformin + Curcumin**	0.654	0.695	0.678	0.980	0.963	0.949
**0.4 µM Carboplatin**	0.985	1.000	1.010	0.988	1.013	0.993
**0.4 µM Carboplatin + Metformin + Curcumin**	0.745	0.699	0.703	0.977	0.991	0.981
**0.4 µM Oxaliplatin**	0.856	0.894	0.885	1.002	1.016	0.987
**0.4 µM Oxaliplatin + Metformin + Curcumin**	0.570	0.565	0.596	1.004	0.995	0.970
**0.037 µM Epirubcin**	0.687	0.631	0.691	0.980	1.025	1.018
**0.037 µM Epirubcin + Metformin + Curcumin**	0.512	0.532	0.543	1.024	1.018	1.029
**0.037 µM Doxorubcin**	0.837	0.786	0.759	0.972	1.021	1.028
**0.037 µM Doxorubcin + Metformin + Curcumin**	0.604	0.583	0.635	1.015	0.985	1.004
**1.2 µM Docetaxel**	0.871	0.937	0.826	0.983	1.007	1.011
**1.2 µM Docetaxel+ Metformin + Curcumin**	0.509	0.552	0.685	1.032	1.040	1.027
**0.4 µM Paclitaxel**	0.539	0.511	0.538	0.983	0.982	0.993
**0.4 µM Paclitaxel + Metformin + Curcumin**	0.393	0.369	0.384	1.000	0.996	0.989
**0.037 µM Methotrexate**	0.463	0.474	0.496	1.001	0.997	0.987
**0.037 µM Methotrexate + Metformin + Curcumin**	0.338	0.367	0.346	1.004	0.981	1.018

*Data of Metformin + Curcumin was used for comparison in each anticancer drug.

EXCLI Journal 2021;20:1488-1498 – ISSN 1611-2156
Received: July 13, 2021, accepted: September 29, 2021, published: October 11, 2021

Raw data of Supplementary Figure 1: Viability of AGS cells after treatment with serial dilutions of anticancer drugs with or without combination of metformin and curcumin for 72 hours. All experiments were carried out independently in triplicate. In each independent biological replication, cell viability was normalized by untreated control cell culture.

Treatment	Replicate	Cisplatin concentration (µM)						
		**0**	**1.2**	**3.7**	**11**	**33**	**100**	**300**
**Cisplatin**	**1**	1	0.912	0.769	0.593	0.135	0.009	0.007
	**2**	1	0.885	0.715	0.539	0.169	0.020	0.001
	**3**	1	0.846	0.736	0.633	0.150	0.024	0.006
**Cisplatin + (0.6 mM Metformin + 0.5 µM Curcumin)**	**1**	1	0.641	0.604	0.471	0.069	0.030	0.023
	**2**	1	0.565	0.535	0.428	0.099	0.004	0.013
	**3**	1	0.599	0.582	0.370	0.077	0.001	0.010

Treatment	Replicate	Carboplatin concentration (µM)						
		**0**	**0.4**	**1.2**	**3.7**	**11**	**33**	**100**
**Carboplatin**	**1**	1	0.994	0.940	0.938	0.617	0.377	0.327
	**2**	1	0.989	0.915	0.877	0.619	0.559	0.343
	**3**	1	0.984	1.028	0.975	0.609	0.502	0.314
**Carboplatin + (0.6 mM Metformin + 0.5 µM Curcumin)**	**1**	1	0.449	0.414	0.406	0.321	0.242	0.139
	**2**	1	0.489	0.481	0.448	0.299	0.246	0.162
	**3**	1	0.492	0.438	0.411	0.295	0.265	0.151

Treatment	Replicate	Oxaliplatin concentration (µM)						
		**0**	**0.4**	**1.2**	**3.7**	**11**	**33**	**100**
**Oxaliplatin**	**1**	1	0.822	0.616	0.443	0.436	0.335	0.083
	**2**	1	0.824	0.607	0.527	0.429	0.327	0.059
	**3**	1	0.927	0.597	0.465	0.466	0.284	0.127
**Oxaliplatin + (0.6 mM Metformin + 0.5 µM Curcumin)**	**1**	1	0.533	0.434	0.340	0.302	0.176	0.026
	**2**	1	0.510	0.449	0.302	0.281	0.196	0.003
	**3**	1	0.474	0.455	0.375	0.313	0.150	0.001

Raw data of Supplementary Figure 1 (cont.)

Treatment	Replicate	Epirubicin concentration (µM)						
		0	0.03	0.11	0.33	1	3	9
Epirubicin	1	1	0.609	0.505	0.295	0.157	0.108	0.098
	2	1	0.568	0.450	0.250	0.155	0.108	0.097
	3	1	0.682	0.470	0.271	0.150	0.110	0.083
Epirubicin + (0.6 mM Metformin + 0.5 µM Curcumin)	1	1	0.416	0.327	0.116	0.031	0.028	0.012
	2	1	0.398	0.294	0.178	0.042	0.035	0.005
	3	1	0.431	0.339	0.114	0.057	0.023	0.014

Treatment	Replicate	Doxorubicin concentration (µM)						
		0	0.03	0.11	0.33	1	3	9
Doxorubicin	1	1	0.561	0.498	0.246	0.012	0.007	0.001
	2	1	0.578	0.518	0.269	0.008	0.010	0.009
	3	1	0.592	0.550	0.259	0.003	0.002	0.000
Doxorubicin + (0.6 mM Metformin + 0.5 µM Curcumin)	1	1	0.374	0.399	0.278	0.026	0.006	0.004
	2	1	0.399	0.383	0.183	0.005	0.016	0.001
	3	1	0.424	0.358	0.244	0.014	0.005	0.007

Treatment	Replicate	Docetaxel concentration (µM)						
		0	1.2	3.7	11	33	100	300
Docetaxel	1	1	0.870	0.658	0.465	0.480	0.357	0.040
	2	1	0.852	0.569	0.487	0.428	0.309	0.092
	3	1	0.884	0.589	0.478	0.414	0.270	0.114
Docetaxel + (0.6 mM Metformin + 0.5 µM Curcumin)	1	1	0.551	0.433	0.320	0.280	0.135	0.000
	2	1	0.544	0.408	0.357	0.370	0.179	0.054
	3	1	0.507	0.458	0.334	0.240	0.198	0.012

EXCLI Journal 2021;20:1488-1498 – ISSN 1611-2156
Received: July 13, 2021, accepted: September 29, 2021, published: October 11, 2021

Raw data of Supplementary Figure 1 (cont.)

Treatment	Replicate	Palitaxel concentration (µM)						
		**0**	**0.4**	**1.2**	**3.7**	**11**	**33**	**100**
**Palitaxel**	**1**	1	0.451	0.426	0.377	0.303	0.052	0.026
	**2**	1	0.502	0.442	0.328	0.328	0.091	0.031
	**3**	1	0.500	0.358	0.298	0.199	0.073	0.011
**Palitaxel + (0.6 mM Metformin + 1 µM Curcumin)**	**1**	1	0.298	0.220	0.169	0.174	0.008	0.009
	**2**	1	0.321	0.278	0.255	0.154	0.002	0.006
	**3**	1	0.264	0.224	0.209	0.143	0.001	0.002

Treatment	Replicate	Methotrexate concentration (µM)						
		**0**	**0.03**	**0.11**	**0.33**	**1**	**3**	**9**
**Methotrexate**	**1**	1	0.380	0.334	0.349	0.367	0.336	0.334
	**2**	1	0.424	0.360	0.327	0.316	0.308	0.302
	**3**	1	0.427	0.366	0.320	0.284	0.288	0.298
**Methotrexate + (0.6 mM Metformin + 1 µM Curcumin)**	**1**	1	0.249	0.251	0.228	0.208	0.190	0.182
	**2**	1	0.279	0.257	0.219	0.198	0.196	0.173
	**3**	1	0.274	0.232	0.211	0.231	0.216	0.203

EXCLI Journal 2021;20:1488-1498 – ISSN 1611-2156
Received: July 13, 2021, accepted: September 29, 2021, published: October 11, 2021

Raw data of Supplementary Figure 2:
Cell viability of AGS and HDF cells treated with anticancer drugs alone and in presence of "Metformin + Curcumin" for 72 hours

Treatments	AGS cells			HDF cells		
	Replicate 1	Replicate 2	Replicate 3	Replicate 1	Replicate 2	Replicate 3
**0.6 mM Metformin**	0.958	0.976	0.987	0.988	0.994	0.974
**1 µM Curcumin**	0.941	0.988	0.974	0.996	0.997	0.972
**Metformin + Curcumin***	0.593	0.656	0.600	1.020	0.954	0.926
**1.2 µM Cisplatin**	0.912	0.885	0.846	0.933	0.915	0.952
**1.2 µM Cisplatin + Metformin + Curcumin**	0.622	0.584	0.599	0.935	0.893	0.925
**0.4 µM Carboplatin**	0.994	0.989	0.984	0.933	0.960	0.997
**0.4 µM Carboplatin + Metformin + Curcumin**	0.449	0.489	0.492	0.966	0.904	0.911
**0.4 µM Oxaliplatin**	0.822	0.824	0.927	0.914	0.946	0.969
**0.4 µM Oxaliplatin + Metformin + Curcumin**	0.533	0.510	0.474	0.942	0.946	0.903
**0.037 µM Epirubcin**	0.609	0.568	0.682	0.984	1.012	0.938
**0.037 µM Epirubcin + Metformin + Curcumin**	0.416	0.398	0.431	0.898	0.924	1.009
**0.037 µM Doxorubcin**	0.561	0.578	0.592	0.998	0.977	1.008
**0.037 µM Doxorubcin + Metformin + Curcumin**	0.374	0.399	0.424	0.917	0.986	0.964
**1.2 µM Docetaxel**	0.870	0.852	0.884	1.015	1.050	0.950
**1.2 µM Docetaxel+ Metformin + Curcumin**	0.551	0.544	0.507	0.937	0.957	0.930
**0.4 µM Paclitaxel**	0.451	0.502	0.500	1.019	0.954	0.967
**0.4 µM Paclitaxel + Metformin + Curcumin**	0.298	0.321	0.264	0.965	0.995	0.992
**0.037 µM Methotrexate**	0.397	0.444	0.427	1.052	1.000	0.928
**0.037 µM Methotrexate + Metformin + Curcumin**	0.249	0.279	0.274	0.928	0.975	0.876

*Data of Metformin + Curcumin was used for comparison in each anticancer drug.

EXCLI Journal 2021;20:1488-1498 – ISSN 1611-2156
Received: July 13, 2021, accepted: September 29, 2021, published: October 11, 2021

Raw data of Table 1:
Investigation of the synergistic effect (Cytotoxicity = 1- viability) of combination treatment of metformin and curcumin on AGS cells. Cell viability inhibition was evaluated after 48 hours of single and combination treatment of metformin and curcumin. Data was analyzed by CompuSyn software.

Treatments	Replicate 1	Replicate 2	Replicate 3	Average*
**Metformin (µM)**				
**625**	0.000	0.005	0.007	0.004
**1250**	0.040	0.014	0.029	0.028
**2500**	0.058	0.065	0.019	0.047
**5000**	0.105	0.049	0.100	0.085
**10000**	0.155	0.206	0.172	0.178
**20000**	0.283	0.228	0.208	0.240
**40000**	0.333	0.415	0.396	0.381
**80000**	0.546	0.510	0.468	0.508
**160000**	0.663	0.658	0.688	0.670
**320000**	0.885	0.869	0.936	0.897
**Curcumin (µM)**				
**0.5**	0.001	0.002	0.000	0.001
**1**	0.000	0.004	0.002	0.002
**2**	0.039	0.025	0.015	0.026
**4**	0.121	0.087	0.021	0.076
**8**	0.218	0.155	0.162	0.178
**16**	0.442	0.402	0.347	0.397
**32**	0.689	0.658	0.597	0.648
**64**	0.855	0.844	0.781	0.827
**128**	0.958	0.949	0.949	0.952
**256**	0.975	0.981	0.981	0.979
**Metformin + Curcumin (µM)**				
**625 + 0.5**	0.268	0.262	0.234	0.255
**1250 + 1**	0.280	0.261	0.262	0.268
**2500 + 2**	0.343	0.286	0.316	0.315
**5000 + 4**	0.368	0.416	0.378	0.387
**10000 + 8**	0.544	0.561	0.586	0.564
**20000 + 16**	0.690	0.758	0.711	0.720
**40000 + 32**	0.841	0.786	0.860	0.829
**80000 + 64**	0.944	0.993	0.956	0.964
**160000 + 128**	0.990	0.930	0.976	0.965
**320000 + 256**	0.983	0.994	0.939	0.972

*Average values were used for analysis.

Raw data of Table 1 (cont.)

Investigation of the synergistic effect (Cytotoxicity = 1- viability) of combination treatment of metformin and curcumin on AGS cells. Cell viability inhibition was evaluated after 72 hours of single and combination treatment of metformin and curcumin. Data was analyzed by CompuSyn software.

Treatments	Replicate 1	Replicate 2	Replicate 3	Average*
**Metformin (µM)**				
**625**	0.042	0.024	0.013	0.026
**1250**	0.069	0.085	0.060	0.071
**2500**	0.073	0.134	0.108	0.105
**5000**	0.100	0.167	0.124	0.130
**10000**	0.267	0.271	0.302	0.280
**20000**	0.424	0.464	0.505	0.464
**40000**	0.619	0.627	0.651	0.632
**80000**	0.676	0.704	0.745	0.708
**160000**	0.805	0.800	0.822	0.809
**320000**	0.932	0.955	0.985	0.957
**Curcumin (µM)**				
**1**	0.059	0.012	0.026	0.032
**2**	0.076	0.045	0.051	0.057
**4**	0.227	0.201	0.153	0.194
**8**	0.374	0.361	0.438	0.391
**16**	0.662	0.633	0.648	0.648
**32**	0.891	0.889	0.907	0.896
**64**	0.982	0.996	0.996	0.991
**128**	0.995	0.995	0.989	0.993
**256**	0.977	0.996	0.998	0.990
**512**	0.997	0.998	0.992	0.996
**Metformin + Curcumin (µM)**				
**625 + 1**	0.407	0.344	0.400	0.384
**1250 + 2**	0.388	0.382	0.410	0.393
**2500 + 4**	0.400	0.403	0.428	0.410
**5000 + 8**	0.437	0.453	0.488	0.459
**10000 + 16**	0.551	0.621	0.573	0.582
**20000 + 32**	0.829	0.837	0.852	0.839
**40000 + 64**	0.925	0.938	0.953	0.939
**80000 + 128**	0.971	0.964	0.994	0.976
**160000 + 256**	0.986	0.992	0.996	0.991
**320000 + 512**	0.989	0.995	0.999	0.994

*Average values were used for analysis.
